# The Association of Periodontitis with Circadian Rhythm Disorders and Sleep—A Scoping Review

**DOI:** 10.3390/medicina62040662

**Published:** 2026-03-31

**Authors:** Mariacristina Amato, Alessandro Polizzi, Marco Mascitti, Angela Angjelova, Elena Jovanova, Andrea Blasi, Gianluca Martino Tartaglia, Gaetano Isola

**Affiliations:** 1 Unit of Periodontology, Department of General Surgery and Surgical-Medical Specialties, School of Dentistry, University of Catania, 95123 Catania, Italy; 2 International Research Center on Periodontal and Systemic Health “PerioHealth”, University of Catania, 95123 Catania, Italy; 3 Department of Clinical Specialistic and Dental Sciences, Marche Polytechnic University, 60126 Ancona, Italy; 4 Department of Periodontology, School of Dental Medicine, University of Naples Federico II, 80131 Naples, Italy; 5 Fondazione Ca’ Granda, IRCCS Ospedale Policlinico, 71013 Milan, Italy; 6 Department of Biomedical, Surgical and Dental Sciences, University of Milan, Via Della Commenda 10, 20122 Milano, Italy

**Keywords:** periodontitis, circadian rhythm, circadian syndrome, sleep, sleep quality, sleep duration

## Abstract

*Background and Objectives*: This scoping review aims to assess the correlation between periodontitis, circadian rhythm disorders, and sleep quality. *Materials and Methods*: This review followed the Preferred Reporting Items for Systematic Reviews and Meta-Analyses extension for Scoping Reviews (PRISMA-ScR) guidelines to identify eligible studies from the following databases: PubMed, Scopus, and Web of Science. The research question was formulated as follows: “What is the evidence about the correlation between periodontitis, circadian rhythm disorders and sleep?”. *Results*: Through database searching, 1673 records were identified, and ultimately, 20 studies were included. Among these, seven articles were about the correlation between circadian rhythm and periodontitis; thirteen articles discussed the association between sleep and periodontitis. *Conclusions*: Circadian rhythm alterations and poor sleep are related to periodontitis. A causal relationship between circadian rhythm disturbances, poor sleep, and periodontitis remains unclear, especially because of the cross-sectional design of most studies. No study has conducted an analysis of the reverse causality. To clarify this correlation, longitudinal studies should be conducted. A few experiments suggested that circadian rhythm disturbances and sleep deprivation induce periodontitis. Assessing direct causality and understanding the precise role of circadian rhythm alterations and sleep quality on periodontitis can lead to personalizing the management of the condition.

## 1. Introduction

Periodontitis is a chronic, multifactorial inflammatory disease affecting tooth-supporting tissues [1], initiated by a negative chronic stimulus from periodontal pathogens, with a multifactorial etiology [2] that, in predisposed individuals, is followed by an unbalanced host response [1] that can cause periodontal tissue damage and an overall lower quality of life. Global incidence and prevalence of periodontitis have increased dramatically over the last few decades, with 50,823,934 cases (95% uncertainty intervals for more than 1.5 billion people (+44.32%)) [3]; by 2050, severe periodontitis is expected to affect more than 1.5 billion people (+44.32%) [4].

Periodontitis is also associated with systemic conditions, including cardiovascular diseases [5,5], diabetes, and neurodegenerative disorders [5,6,7,8,9,10]. The progression of periodontitis can be influenced by co-factors, including the above-mentioned diseases [11,12,13], bad habits, such as smoking [14,15], and lack of physical activity [16]. Considering such aspects during the periodontal risk assessment helps in the management of periodontitis, giving the opportunity to tailor the therapy and to obtain a favorable prognosis [17].

On the other hand, circadian rhythm is a survival instinct of every organism that enables it to predict and prepare for predictable environmental variations determined by the daily rotation of the Earth [18,19]. Regular physiological activity is regulated by the circadian clock, which is present in all cells and organs of mammals [20,21]. It is a group of circadian genes that regulates the expression of genes responsible for cell physiology and metabolism [22,23]. Nowadays, circadian rhythm disorders are widespread across populations, since they are related to stressful shift work, sleep disorders, and various inflammatory diseases [24,25]. In some cases, individuals are diagnosed with circadian syndrome (CircS), which is defined as a group of conditions, including metabolic syndrome (MetS), short sleep duration (<6 h/day), and depression symptoms (assessed via the ten-question version of the Center for Epidemiologic Studies-Depression scale [CES-D]) [26,27,28]. The presence of circadian rhythm disorders often aggravates pre-existing diseases and causes the production of inflammatory factors [29]. Since periodontitis is an inflammatory condition and is strictly related to pathologies influenced by circadian disorders, like cardiovascular diseases [26], circadian disturbances may be related to it too [30]. In addition, as already mentioned, sleep is related to circadian rhythm [31], and sleep disturbances have been suggested to be related to oral health and to periodontitis too [32,33,34].

### Aim

Considering the above-mentioned evidence, the aim of this scoping review is to assess the correlation between periodontitis, circadian disorders, and sleep.

## 2. Materials and Methods

### 2.1. Study Design

In order to identify the research question, to select the appropriate studies, to establish inclusion and exclusion criteria, to extract data, and to summarize the results, the present review follows the PRISMA-ScR (Preferred Reporting Items for Systematic Reviews and Meta-Analyses) [35] extension for scoping reviews (Supplementary File S1).

### 2.2. Objective and PCC Framework

This scoping review aimed to explore the existing evidence about the association between periodontitis and circadian rhythm and/or sleep in humans and animals.

The review was guided by the Population–Concept–Context (PCC) framework:

Population (P): Humans affected by periodontitis, humans affected by CircS, humans with low quality of sleep, humans with circadian rhythm disorders/circadian rhythm disruption (CRDs/CRD), animal models of periodontitis, animal models of periodontitis with CRDs/CRD, and animal models of periodontitis with sleep deprivation.

Concept (C): Chronic periodontitis, severe periodontitis, induced periodontitis, CircS, CRD, CRDs, sleep, sleep quality, sleep duration, sleep deprivation, and inflammatory molecules.

Context (C): Experimental and clinical settings investigating the prevalence of the association of periodontitis with circadian rhythm alterations and periodontitis with quality and duration of sleep.

### 2.3. Literature Search

The literature search of articles was performed in three scientific search engines, including PubMed, Web of Science, Scopus, and the Cochrane Library, using the refined search strategy developed after choosing the appropriate keywords for the research question with consistent Boolean operators (‘AND’ and ‘OR’). The research question was formulated as follows: “What is the evidence about the correlation between periodontitis, circadian rhythm disorders and sleep?”. The full search strategy (Supplementary Table S1) consisted of free text words combined with MeSH terms through Boolean operators, covering all years to date. It was carried out in February 2026 to find all manuscripts related to the relationship of periodontitis with circadian rhythm and with sleep. Furthermore, additional articles were retrieved manually from Google Scholar and the reference list of the eligible studies to include potential additional reviews.

### 2.4. Inclusion and Exclusion Criteria

In this scoping review, exact inclusion and exclusion criteria were used to guide the selection of the articles (Table 1).

In particular, publications written in English were only accepted if they discussed periodontitis and circadian rhythm and/or sleep. Manuscripts with the following study designs were taken into account: cross-sectional studies, experimental animal studies, experimental human studies, cohort studies, case–control studies, randomized and non-randomized controlled clinical trials, and retrospective investigations. Conversely, all articles that did not analyze the relationship of periodontitis with circadian rhythm or with sleep were not fully textually available in English, and included certain study designs (such as opinion pieces, theses, conference reports, case reports, case series, and any type of review articles) were not included.

### 2.5. Study Selection

The selection of the studies involved two separate authors, who used EndNote^TM^ 20desktopversion (Clarivate, 1500SpringGardenStreet, FourthFloor, Philadelphia, PA 19130, USA, October 2020) for the screening and selection of relevant studies [36].

The first analysis of the articles concerned titles and abstracts, after duplicates were eliminated. Every article that met the inclusion criteria had its full-text version examined in more detail. If there was any controversy following the comparison of the final findings, a third researcher was consulted. The article and the year of publication, the aim, the sample, the study’s design, the indication, and the results were all extracted from the papers included.

## 3. Results

**Figure 1:**
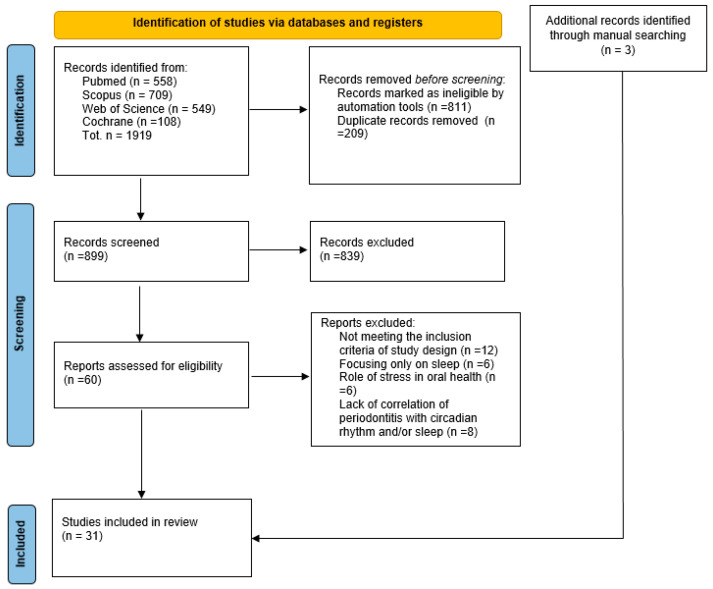
PRISMA-ScR flow diagram of the articles’ identification.

The search strategy led to the identification of a total of 1919 records in the PubMed (n = 558), Scopus (n = 709), Web of Science (n = 549), and Cochrane (n = 108) databases (Table 1). Next, 811 records were marked as ineligible by automation tools; then, 209 duplicates were removed, and the remaining 899 records were screened for titles and abstracts (Figure 1).

Subsequently, 839 records were excluded, and 60 papers were assessed for eligibility through full-text review. After meticulous analysis, twelve articles were excluded because they did not meet the inclusion criteria about the study design, six studies were excluded because they focused only on sleep, six articles were excluded because they discussed the role of stress in oral health, and eight manuscripts were excluded because they did not discuss the correlation between circadian rhythm and periodontitis or sleep and periodontitis. A total of three manuscripts were obtained through a manual search on Google Scholar. Therefore, at the end of the selection process, 31 articles were included in this scoping review.

### 3.1. Periodontitis and Circadian Rhythm

In Table 2, eight articles regarding the correlation between periodontitis and circadian rhythm have been included and summarized. Regarding the study design, three papers were cross-sectional [35,37,38], two articles had both a cross-sectional analysis and an experimental part [39,40], and the remaining three manuscripts were experimental studies [30,41,42]. Some articles analyzed how periodontitis influenced circadian rhythm gene pathways in periodontal tissues [39,41]; other papers evaluated the influence of circadian rhythm syndrome on periodontitis [30,35,37,38,40,42].

### 3.2. Periodontitis and Sleep

In Table 3, 23 articles regarding the correlation between periodontitis and sleep have been included and summarized. Regarding the study design, nineteen papers were cross-sectional [34,44,45,46,47,48,49,50,51,52,53,54,55,56,57,58,59,60,61], two articles were experimental studies [62,63], and two manuscripts were case–control studies [64,65]. Some articles showed how insufficient sleep duration and poor sleep quality were associated with periodontitis [34,50,52,55,56,57,61,64,66], and that larger sleep duration was a protective factor against periodontitis, especially for patients affected by diabetes [45]. In contrast, one study [47] detected that people who slept more were more likely to be affected by periodontitis, and in another study [48], such association was true only for women. Other studies did not find any associations between sleep duration and sleep quality [49,58,60]; one study [59] suggested a direct and independent link between abnormal sleep duration and periodontitis. The two experimental studies showed that sleep deprived experimental mice developed severe periodontitis [62,63].

## 4. Discussion

This scoping review aims to provide a panoramic view of the current state of knowledge about the association of circadian rhythm, circadian rhythm alterations, and sleep quality and quantity with periodontitis. The results of the present review show that such correlations exist; thus, they may allow us to better understand the mechanisms underlying them. In this way, when making the evaluation of a periodontitis patient, considering sleep quality and quantity and the circadian rhythm of the patient gives the possibility to personalize the management of the disease.

### 4.1. Circadian Rhythm and Periodontitis

Circadian rhythm is a daily biological cycle that controls a wide range of physiological processes, including sleep [67]. Both natural and artificial light influence circadian rhythm through endogenous oscillators, which include neural, hormonal, and genetic elements [68]. Circadian system functions are managed by central and peripheral oscillators. Such peripheral oscillators are controlled by the cyclical expression of clock genes [69]. Clock genes encode transcription factors called clock proteins, whose concentration levels oscillate in a cyclic way [69]. This leads to the activation of specific processes at predetermined time points. Periodontal tissues have clock genes, the expression of which produces clock proteins that regulate the biological processes in the periodontal environment [70,71,72], which are surely impacted by periodontal treatment [73,74,75].

#### 4.1.1. Experimental Studies About Circadian Rhythm and Periodontitis

Some of the reported experimental studies have investigated the expression of clock genes under periodontitis. In particular, Ebersole et al. [39] demonstrated that periodontitis and aging caused a reduction in clock genes *N CLOCK*, *PER1-3*, *RORA*, *NR1D1*, and *ID2* expression in periodontal tissues in monkeys. In another study, Guo et al. [41] showed alterations in circadian genes in gingival tissues in periodontitis rat models and detected an increase in inflammatory factors in the periodontal environment. Such discoveries suggest that an inflammatory condition is related to the variation in the expression of clock genes. The core clock gene BMAL1 was found to be downregulated during CRD and periodontitis in rats, suggesting a central role of such a gene in the regulation of inflammation, oxidative stress, and bone metabolism in periodontal tissues [42]. Ma et al. [30] assessed the correlation between CRD and periodontitis and detected that CRD impaired macrophage homeostasis, promoting pro-inflammatory M1 polarization and reducing anti-inflammatory M2 macrophages. They also observed elevated levels of pro-inflammatory cytokines (e.g., TNF-α, IL-1β) and oxidative stress in periodontal tissues.

Identifying the clock genes involved in circadian rhythm disruption helps in better understanding the molecular mechanisms underlying the inflammation, which leads to periodontal damage. Therefore, if validated, the research on expressions of such genes may be useful in diagnosing periodontitis, and those genes may become the target of personalized periodontal therapy. All these studies were conducted in animals; thus, such discoveries need to be tested in humans too.

#### 4.1.2. Human Cross-Sectional Studies About CircS and Periodontitis

**Figure 2:**
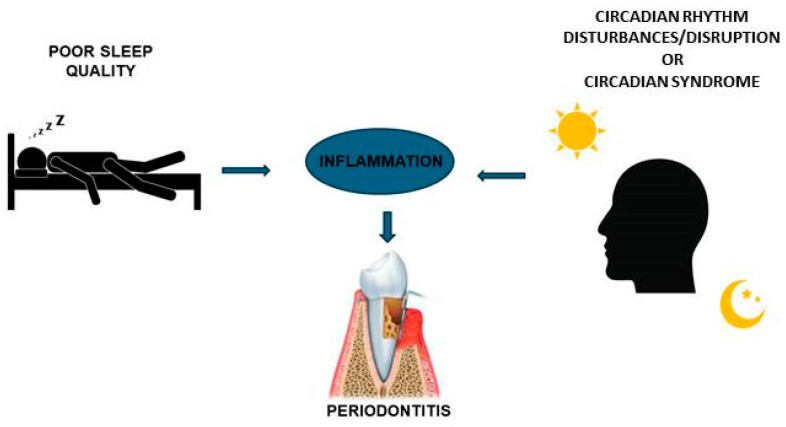
Circadian rhythm and sleep are related to periodontitis. Alterations in normal circadian rhythm (circadian rhythm disturbances/disruption or circadian syndrome) and poor sleep quality induce inflammatory conditions, which are suggested to lead to periodontitis.

Other studies conducted human cross-sectional analysis about the correlation of CircS and periodontitis. CircS is a group of metabolic, cardiovascular, and sleep-related disturbances linked to circadian rhythm disruption. It alters homeostasis, induces oxidative stress, promotes an inflammatory response, and accelerates the coagulation process [76,77,78]. People affected by CircS are more susceptible to developing hypertension, diabetes mellitus (DM), hyperlipidemia, obesity, atherosclerosis, and other inflammatory diseases, including periodontitis [76,79,80,81,82]. Li et al. [35] have examined data from U.S. NHANES from 2005 to 2020 of 15,966 individuals aged 30–85 years, detecting that individuals affected by periodontitis or with greater tooth loss and poor oral health were associated with higher odds of CircS (odds ratio (OR)) and a 95% confidence interval (CI): stage II: 1.35 (1.03, 1.76), *p* = 0.032; stage III: 1.30 (0.97, 1.73), *p* = 0.069; and stage IV: 1.17 (0.82, 1.65), *p* = 0300). Zhang et al. [37] conducted a cross-sectional analysis of U.S. NHANES from 209 to 2014 of 7555 participants and evaluated the association between CircS and periodontitis. They confirmed that CircS and periodontitis are significantly correlated, even after adjusting for confounders (odds ratio 1.509, 95% CI 1.326–1.716, *p* < 0.0001) (Figure 2).

However, the number of CircS components showed a nonlinear association with periodontitis, present only when components were <4, with no further increase after diagnosis of CircS. This relationship was partly mediated by serum lipids, oxidative stress, and inflammation markers. The association was stronger in individuals under 60 years old, with higher income, and those who were physically inactive. These findings suggest considering CircS screening in periodontal risk assessment, particularly in these groups. Nevertheless, the reported studies that assessed the association of CircS and periodontitis had cross-sectional designs, which cannot exclude reverse causality; for this reason, before introducing CircS diagnosis in the evaluation of periodontitis, it is necessary to validate it through longitudinal studies.

### 4.2. Sleep and Periodontitis

On the other hand, some researchers have also investigated the correlation between sleep and periodontitis, focusing on sleep time, sleep duration, and sleep quality [44,45,46,47,48,49,50,51,51,52,53,54,62,63]. Sleep has been investigated from multiple perspectives among the reported manuscripts. Some studies have evaluated sleep quality, which embraces multiple aspects of sleep, including subject perception of quality of sleep, sleep duration, presence of sleep disturbances (breathing and non-breathing sleep disorders), and how restorative the sleep is [83,84]; these are typically assessed using standardized scales, such as PSQI and Jenkins Sleep Scale [46,49,50,52]; Han et al. [47] have taken into account sleep time, which indicates the moment at which each participant goes to sleep; in some studies, sleep duration has been taken into account and short and long sleep have been defined arbitrarily based on the number of hours slept by participants [44,45,48,49,53]; finally, the experimental animal study of Nakada et al. [63] has investigated the impact of sleep deprivation, which refers to a total absence of sleep.

Sleep is one factor that most strongly influences the circadian clock genes and leads to CRD or CircS [85]; these conditions have been demonstrated to induce inflammatory status and to be correlated with periodontitis [35,37]. Thus, sleep may have a central role in a lot of conditions and diseases, including periodontitis (Figure 2) [33].

#### 4.2.1. Human Cross-Sectional Studies About Sleep and Periodontitis

Most of the existing studies have investigated the association between sleep duration and the prevalence of periodontitis by conducting cross-sectional analysis of data, including that from U.S. NHANES [51], from Korean NHANES [48], and data collected at a university in Japan [49]. They assessed that sleep deficiency (<7 h) was significantly associated with periodontitis. They also investigated which relationship excessive sleep had with periodontitis, giving the following results: for Alhassani et al. [44], no statistically significant association between longer sleep duration and periodontitis was revealed; in contrast, Han et al. [48] showed that longer sleep duration was associated with the highest ratio of periodontitis in women, especially in premenopausal women, suggesting the presence of hormonal factors too. The same group [47] detected a higher risk of periodontitis in individuals who slept during the daytime and who slept >9 h, but also demonstrated that systemic inflammation has a mediating role, decreasing the strength of such an association. In addition, Liu et al. [51] confirmed the correlation between reduced sleep duration and periodontitis but also the nonlinear association of periodontitis with sleep duration, indicating other systemic factors, such as hypertension, as mediators for developing periodontitis. According to Islam et al. [49], no association with sleep was demonstrated; in fact, they recognized as a main causing factor of periodontitis only poor oral hygiene and did not find any correlation between sleep and periodontitis. Most of these studies, especially those that analyzed the data from NHANES, had very large and various samples, which may allow for generalizable results. However, considering the cross-sectional study design, which limits the power of correlation, it would be ideal to conduct longitudinal studies in order to validate or refute such discoveries. Moreover, studies had controversial results; thus, it is necessary to better understand the nature of the relationship between sleep and periodontitis and possible other involved factors, including systemic inflammation or other pathologies, as suggested by some authors [51].

#### 4.2.2. Case–Control Studies About Sleep and Periodontitis

Even if the majority of the studies have a cross-sectional design, there are also studies that have investigated the association of periodontitis with sleep using a case–control study design. In particular, two of the reported studies [64,65] compared sleep characteristics among periodontitis patients and healthy controls. The results assessed the existence of a correlation, since in both studies, poor sleep quality was associated with periodontitis. Nevertheless, the sample size of these studies was limited; thus, in order to assess such a correlation, bigger samples are needed.

#### 4.2.3. Experimental Studies About Sleep and Periodontitis

In addition to the human cross-sectional and case–control studies, the literature does not lack in experimental animal studies. In particular, in the study of Nakada et al. [63], rats subjected to sleep deprivation developed more severe periodontal destruction than those in the control group. High levels of systemic inflammation were detected, indicating that fatigue from insufficient sleep may impair the ability of the body to regulate the periodontal inflammatory process. These results find confirmation in the above-mentioned human cross-sectional studies [44,45,46,50,51,51,52]. On the other hand, Li et al. [62] conducted an experimental study, which involved both humans and rats. They showed that in both clinical subjects and animal models, sleep deficiency was associated with increased risk and severity of periodontitis. In mice, TRPV1-expressing trigeminal neurons were found to connect the nervous system to periodontal tissues. Sleep restriction increased the release of substance P, leading to vasodilation, higher vascular permeability, and infiltration of inflammatory cells. Ablation of these neurons prevented periodontitis, indicating their key role in periodontitis pathogenesis. Thanks to the results of this study, new insights about the mechanism underlying the association between sleep and periodontitis were revealed; however, it is necessary to investigate more by conducting other new studies.

### 4.3. Interpretation of Findings

The reported studies suggest the existence of a biological and epidemiological relationship between circadian rhythm disruption and periodontitis, although the strength and nature of this association vary depending on the study design. Experimental animal studies [30,39,40,41,42] provide evidence showing that circadian disruption alters the expression of core clock genes (e.g., BMAL1, CLOCK, PER) and promotes periodontal inflammation, bone loss, oxidative stress, and immune dysregulation. These findings suggest the existence of a biological pathway in which circadian imbalance causes periodontal tissue damage, through increased osteoclast activity and pro-inflammatory signaling. On the other hand, cross-sectional human studies [37,38] report a significant association between circadian syndrome and periodontitis. Some studies also suggest potential mediating factors, such as systemic inflammation, oxidative stress, and metabolic dysfunction, which need to be further investigated, too, in order to assess potential multifactorial interactions. Animal models suggest a directional effect of circadian disruption on periodontal disease progression; however, the predominance of human cross-sectional designs limits causal inference; for this reason, further longitudinal and interventional studies are needed to establish causality. The relationship between sleep disturbances and periodontitis has been widely investigated, but current evidence is very heterogeneous, with findings that are not entirely consistent. The majority of cross-sectional and case–control studies [44,45,52,56,57,64,65] report a significant association between poor sleep quality or altered sleep duration and increased prevalence or severity of periodontitis, with CAL, worse periodontal parameters, or increased odds of periodontitis in sleep-deprived adults. Metabolic conditions, or shift work, suggests a multifactorial relationship. Nevertheless, other investigations [49,54,58,60] fail to confirm this association or indicate that sleep parameters alone may not be sufficient predictors of periodontal disease. Experimental animal studies [62,63] support a biological link, showing that sleep deprivation exacerbates inflammation and periodontal destruction through neuro–immune and inflammatory pathways. It is important to highlight that the Mendelian randomization analysis [61] did not demonstrate a causal relationship, strengthening the limitation of observational evidence. Thus, a consistent trend toward an association is observed, but the variability in methodologies, populations, and definitions, together with the predominance of cross-sectional designs, makes it difficult to establish causality and highlights the need for longitudinal and interventional studies.

### 4.4. Limitations and Future Perspectives

The present scoping review highlighted the potential correlation between periodontitis, sleep disorders, and circadian rhythm disruption. However, some limitations must be acknowledged. First of all, according to the findings, a high heterogeneity in study design should be pointed out. For instance, animal models, cross-sectional epidemiological studies, mechanistic molecular studies, and small pilot studies were all included. Such differences require caution when interpreting the results presented in this scoping review, since high variability in methods and study designs limited the level of evidence. Furthermore, the lack of long-term longitudinal studies prevents one from establishing an unequivocal and direct causal link. The majority of the reported articles assessed the existence of a correlation between the circadian rhythm alteration and bad quality of sleep with periodontitis. However, a limitation is represented by the cross-sectional design of most studies; in fact, in such cases, it is not possible to assess which condition causes the other. Another limitation is represented by the heterogeneity of definitions of periodontitis and sleep parameters across studies. In fact, in some cases, periodontitis is defined according to the 2017 classification of periodontitis [2]; in others, it is defined using CPI criteria [86,87]. Sleep parameters vary too; the definition of poor sleep is not standardized among studies, but each study has given its own definition and duration time. This heterogeneity limits the power and the reliability of the results. Moreover, most of the existing studies, especially the experimental animal and human studies, only analyze whether circadian rhythm alterations or poor sleep may cause periodontitis and not the reverse. For this reason, in order to bypass the limits of the cross-sectional design and to assess which condition causes or influences the other, longitudinal human studies analyzing whether periodontitis influences circadian rhythm and sleep quality need to be conducted. In addition, in order to homogenize the results, standardized periodontal and sleep parameters need to be employed.

## 5. Conclusions

Most of the current studies assess that circadian rhythm disruption and poor sleep are associated with an increased risk of periodontitis, although a direct causal relationship cannot be established, mainly due to the predominance of cross-sectional study designs and the lack of analyses on reverse causality. There is evidence that suggests that this association may be partially mediated by systemic inflammatory conditions. Experimental findings provide preliminary insights into the biological mechanisms linking sleep deprivation to periodontal inflammation, but further research is needed to confirm and expand these results. Therefore, longitudinal studies are essential to clarify causality and directionality. These conclusions should be considered exploratory due to the current limitations of the available evidence. Once the role of circadian rhythm alterations and sleep quality in periodontitis is better understood, it may help improve periodontal risk assessment and lead to a more personalized approach to periodontitis management.

## Figures and Tables

**Table 1:** Inclusion and exclusion criteria.

Domain	Inclusion Criteria	Exclusion Criteria
Language	English	Other languages
Topic	Periodontitis and circadian rhythm Or Periodontitis and sleep	Articles not discussing association of periodontitis with circadian rhythm or sleep
Study design	cross-sectional studies, experimental animal studies, Experimental human studies, cohort studies, case–control studies, randomized and non-randomized controlled clinical trials, and retrospective investigations.	Opinion pieces, theses, conference reports, case reports, case series, and any type of review articles
Availability	Full text	Only title and abstract

**Table 2:** Circadian rhythm, circadian clock genes, and periodontitis.

Study	Aim	Study Design	Sample Size	Materials and Methods	Main Results
Ebersole et al. [39]	To examine how circadian rhythm pathway genes in gingival tissues are affected by aging and by periodontitis.	Cross-sectional and experimental animal study	34 *Macaca mulatta* monkeys for the cross-sectional model and 34 *Macaca mulatta* monkeys for the experimental model	Periodontitis was experimentally induced by placing ligatures around selected teeth and assessed by BOP and PPD > 4 mm. Tissue samples were collected under sedation at baseline, during disease, and after resolution. Microarray data (ArrayExpress E-MTAB-1977; GEO GSE180588) were analyzed for fold-changes in gene expression compared to healthy adult tissues. Statistical and correlation analyses determined associations with inflammation, bone metabolism, and hypoxia-related genes.	Aging and periodontitis both significantly altered the expression of circadian rhythm genes. In healthy young and aged tissues, several clock genes (e.g., NPAS2, NR1D1, RORA) were downregulated compared with healthy adults. In diseased tissues, many circadian genes were further reduced, including CLOCK, PER1-3, RORA, NR1D1, and ID2, while FOS was increased. During ligature-induced disease, reductions in circadian gene expression appeared early and persisted even after clinical resolution.
Guo et al. [41]	To investigate how periodontitis affects the expression and rhythmicity of circadian clock genes in mouse alveolar bone.	Experimental animal study	50 C57 mice, divided into two groups: a periodontitis group and a healthy control group.	Micro-CT scanning measured bone height and attachment loss. qRT-PCR quantified mRNA expression of circadian genes (*Bmal1*, *Per2*, *Cry1*) and inflammatory factors (IL-6, TNF-α, IFN-γ).Statistical comparisons were made between the periodontitis and control groups, and rhythmicity of gene expression was analyzed.	Mice with periodontitis showed reduced alveolar bone height and increased levels of inflammatory cytokines (IL-6, TNF-α, IFN-γ). While healthy mice displayed normal circadian expression of *Bmal1*, *Per2*, and *Cry1*, this rhythmic pattern was lost in periodontitis, indicating that inflammation may disrupt circadian regulation in alveolar bone.
Han et al. [38]	To investigate the association between CircS and periodontitis in U.S. adults, and to identify high-risk subgroups for targeted prevention.	Cross-sectional study	5343 U.S. adults	Data from NHANES 2009–2014 were used. Periodontitis was defined by PPD and CAL as proposed by Eke et al. [43]; CircS required ≥ 4 of 7 components (waist circumference, triglycerides, HDL-C, BP, fasting glucose, sleep ≤ 6 h, PHQ-9 ≥ 5). Weighted multivariable logistic regression, subgroup analyses and PSM were used.	CircS was associated with 29% higher odds of periodontitis (OR = 1.29, 95%CI: 1.08–1.53). Subgroups with elevated risk included females (OR = 1.499), non-Hispanic Whites (OR = 1.352), unmarried individuals (OR = 1.367), non-smokers (OR = 1.308), and those with low education (OR = 1.880).
Li et al. [35]	To examine the associations between periodontitis, tooth loss, and self-rated oral health and the presence of CircS among U.S. adults.	Cross-sectional study	15,966 U.S. adults aged 30–85	NHANES data from U.S. adults were used to examine the association between CircS and oral health. CircS was defined as a cluster of metabolic, cardiovascular, and sleep disturbances related to circadian disruption. The study analyzed periodontitis, classified according to the 2017 periodontitis classification, tooth loss, and self-rated oral health using adjusted multivariable logistic regression.	Individuals with periodontitis or greater tooth loss were significantly more likely to have circadian syndrome. Poor self-rated oral health was also associated with higher odds of CircS. These associations remained significant after adjusting for confounders such as age, sex, BMI, smoking, and socioeconomic status.
Liu et al. [42]	To investigate how CRDs influence the progression of periodontitis, focusing on the role of the core clock gene BMAL1.	Experimental animal study	32 rats	Researchers used rat model of periodontitis combined with circadian rhythm disruption induced by a MMPM. BMAL1 expression levels, alveolar bone resorption, osteoclast differentiation, NF-κB activation, oxidative stress markers, and apoptosis were analyzed in periodontal tissues	BMAL1 expression was significantly downregulated in rats with CRDs and periodontitis. These rats showed increased alveolar bone loss, enhanced osteoclast differentiation, and elevated NF-κB signaling, indicating stronger inflammatory responses. Oxidative stress and apoptosis levels were increased in periodontal tissues under CRDs conditions.
Ma et al. [30]	To explore how circadian rhythm disorder influences the progression of periodontitis, with a focus on its effects on macrophage function and alveolar bone loss.	Experimental animal study	24 male C57BL/6 mice	An induced mouse model of periodontitis (2 weeks of LPS injection and silk ligature placement) with circadian disruption was used to assess bone changes, macrophage polarization, inflammatory cytokines, and immune-related pathways. In vitro studies on macrophages further examined inflammation and oxidative stress under circadian disruption.	Mice with circadian rhythm disruption showed increased alveolar bone loss, heightened periodontal inflammation, and elevated pro-inflammatory cytokines and oxidative stress. This condition promoted M1 macrophage polarization and reduced M2 cells, with underlying dysregulation of circadian clock genes and inflammatory pathways contributing to tissue damage.
Ye et al. [40]	The study aimed to assess whether CRD exacerbates alveolar bone loss and inflammation in rat models of apical periodontitis, focusing on the role of the clock gene BMAL1, and to evaluate its impact in humans with the same condition.	Experimental animal study and cross-sectional study (humans)	32 male Sprague–Dawley rats, divided into 4 groups. BMAL1-deficient cohort of 12 rats (6 knockout + 6 wild-type). 113 human patients	In patients with apical periodontitis, melatonin and cortisol levels were measured to assess the link between circadian disruption and disease severity. In parallel, a rat model with induced periodontitis and circadian disruption was used to evaluate bone damage, inflammation, and BMAL1 expression, along with experiments on BMAL1 deficiency and melatonin treatment to investigate mechanisms and therapeutic potential.	Patients with apical periodontitis and circadian disruption showed altered melatonin and cortisol levels, linked to greater disease severity. In rats, circadian disruption worsened bone loss, inflammation, and osteoclast activity, while BMAL1 expression was reduced. BMAL1 deficiency further increased tissue damage; melatonin treatment partially restored circadian balance and reduced bone resorption.
Zhang et al. [37]	To explore the association between CircS and periodontitis, and to determine whether serum lipids, oxidative stress, and systemic inflammation act as mediating mechanisms.	Cross-sectional, population-based study	7015 adults	Data of participants of NHANES ≥ 20 years old with complete data on periodontal status and CircS components were evaluated. Periodontitis was assessed according to the CDC/AAP criteria. Weighted multivariable logistic regression, restricted cubic spline models, mediation analysis (biomarkers: lipids, vitamin D, albumin, uric acid, CRP, WBC, neutrophils), and subgroup analyses were conducted.	CircS was significantly associated with higher odds of periodontitis (fully adjusted OR = 1.509, 95% CI 1.326–1.716, *p* < 0.0001).

AP: Apical periodontitis; BMI: Body Mass Index; BOP: Bleeding on Probing; CDC/AAP: Centers for Disease Control and Prevention/American Academy of Periodontology; CRD: Circadian rhythm disruption; CRDs: Circadian rhythm disorders; CircS: Circadian Syndrome; CRP: C-reactive protein; MMPM: Modified multi-platform method; PPD: Probing Pocket Depth; WBC: White Blood Cells.

**Table 3:** Sleep and periodontitis.

Study	Aim	Study Design	Sample Size	Materials and Methods	Main Results
Ajikumar et al. [65]	To investigate the relationship between sleep quality and chronic periodontitis, based on their shared inflammatory background.	Case–control study	198 participants	Data collection included demographic information and an interviewer-administered questionnaire to assess sleep quality using the PSQI. Periodontal status was evaluated clinically using the CPI according to WHO criteria. Participants were then categorized, and statistical analysis was performed to assess the association between sleep quality and periodontal health.	Sleep-deprived patients showed greater CAL, ranging from 3 to 5 mm to more than 12 mm. The odds ratio indicated that individuals with periodontitis had 3.36 times higher risk of sleep deprivation compared to those without periodontitis. Poor sleep quality was identified as a significant risk factor for periodontitis.
Alhassani et al. [44]	To investigate the association between sleep duration and the prevalence of periodontitis in a large, nationally representative U.S. sample.	Cross-sectional observational study	10,291 participants aged ≥ 30 year	NHANES 2009–2014 data exams were analyzed. Sleep was self-reported and categorized (<7, 7–8, >8 h), and periodontitis was defined using CDC/AAP criteria. Weighted logistic regression, adjusted for demographic and lifestyle factors, was used to evaluate the association between periodontitis and sleep duration.	5244 participants had periodontitis. In the fully adjusted model, sleep deficiency (<7 h) was significantly associated with increased odds of periodontitis (OR ≈ 1.19), while the association with excessive sleep (>8 h) was not statistically significant.
Alqaderi et al. [45]	To evaluate the association between sleep duration and severe periodontitis in a representative sample of U.S. adults, and to assess whether diabetes modifies this association.	Cross-sectional observational study	3624 U.S. participants aged ≥ 30 years	Data from NHANES 2013–2014 were analyzed. Sleep duration and trouble sleeping were self-reported. Severe periodontitis was defined based on periodontal exam data from NHANES as an individual having ≥2 interproximal sites with ≥6 mm of loss of attachment not on the same tooth, and ≥1 interproximal sites with probing depths of ≥5 mm. Weighted multivariable logistic regression was used for the association between sleep and severe periodontal disease. Effect modification by diabetes was studied too.	People who slept > 7 h/night and reported no trouble sleeping had about 40% lower odds of having severe periodontitis (OR = 0.6, *p* < 0.05), after adjustment. In people with diabetes, the protective association of sleep was stronger (OR~4.8 for the interaction).
Beydoun et al. [54]	To examine the relationships among periodontal disease, self-reported sleep duration, and white blood cell (WBC) markers in a nationally representative sample of U.S. adults.	Cross-sectional observational study	11,813 U.S. participants aged 30 to 80 years	NHANES 2009–2014 data were analyzed to examine the relationships between sleep duration, periodontal disease, and WBC markers. Periodontal status was clinically assessed according to CDC/AAP criteria, sleep duration was self-reported, and WBC counts were obtained from blood tests. Adjusted statistical models, including regression and structural equation modeling, were used to evaluate direct and indirect associations.	Periodontal disease affected about 49.8% of participants, and one-third reported sleeping less than 7 h. Sleep duration was not significantly associated with periodontal disease or WBC markers. However, periodontal disease was linked to higher WBC counts, increased neutrophils, and reduced lymphocytes (especially in men). Sleep duration modified some of these associations but did not mediate the relationship between periodontal disease and WBC markers.
Devarajan et al. [55]	To analyze a potential link between quality of sleep and periodontitis.	Observational cross-sectional study	75 participants, divided into three groups (n = 25 each): periodontitis, gingivitis, and healthy controls.	Periodontal status was clinically assessed. Sleep quality was evaluated using questionnaires (e.g., Berlin and Pittsburgh scores). Salivary samples were collected to measure melatonin levels.	A significant association between sleep and periodontal status was observed. Melatonin levels varied with disease severity, and worse sleep scores were associated with more severe periodontal conditions. No significant association was found in healthy individuals.
Eroglu et al. [46]	To evaluate the association between periodontitis and quality of sleep.	Cross-sectional pilot study	93 periodontitis patients, 31 healthy controls	Adults (18–65 years) with healthy gingiva or untreated periodontitis were included. Periodontal status was clinically assessed and classified by stage and grade according to the 2017 periodontal classification. Participants completed questionnaires about oral hygiene; sleep quality was assessed using (PSQI) and Jenkins Sleep Scale, fatigue was assessed by the Multidimensional Assessment of Fatigue, and oral-health–related quality of life was assessed by the OHIP-14. Statistical analyses included t-tests, chi-square tests, ANOVA, and correlation tests (*p* < 0.05).	Significant shorter sleep duration in the group of patients affected by stage IV periodontitis compared to other groups was observed. Even though sleep duration decreased as both stage and grade of periodontitis increased, the relationship with grade did not reach statistical significance. When sleep quality and fatigue scores were compared between periodontitis patients and healthy controls no statistically significant differences were noticed.
Grover et al. [56]	To assess if there is an association of sleep quality. with chronic periodontal diseases.	Observational cross-sectional study	60 participants	Participants were categorized into the following 3 groups (n = 20 each): clinically healthy, gingivitis and periodontitis. Periodontal status of subjects was assessed by gingival index and PPD. All the study subjects were administered PSQI questionnaire for the assessment of sleep deprivation.	Mean PSQI was highest in the periodontitis group as compared to other two groups and the difference among three groups was statistically significant.
Han et al. [47]	To evaluate the association between sleep duration, sleep time with periodontitis in a cohort of Koreans.	Cross-sectional observational study	4407 participants aged 45–64 years	Periodontal status was assessed using the CPI, defining periodontitis as CPI ≥ 3 and severe periodontitis as CPI = 4. Sleep time and sleep duration were self-reported. Covariates were included in adjusted models. This study used multivariable logistic regression to examine associations between sleep characteristics and periodontitis, and additional models incorporated with white blood cell (WBC) count to explore potential mediation by systemic inflammation.	Daytime sleep time was significantly associated with periodontitis (OR = 1.49; 95% CI: 1.07–2.07). Individuals who went to bed at night but slept ≥9 h were significantly associated with periodontitis (OR = 1.69; 95% CI: 1.04–2.77) and severe periodontitis (OR = 1.88; 95% CI: 1.02–3.45). WBC count in the analysis reduced the strength of some associations.
Han et al. [48]	To assess the association between long sleep duration and periodontal disease among men and women of Korea.	Cross-sectional observational study	14,675 participants aged 19 years or older	Data from 2012 to 2014 from the Korean National Health and Nutrition Examination Survey were analyzed. CPI was used to define periodontitis, with CPI ≥ 3 indicating periodontitis. Sleep duration was self-reported and categorized as follows: “short sleep” (≤5 h), “reference sleep” (6–8 h), and “long sleep” (≥9 h). The association between sleep duration and periodontitis was evaluated by multivariable logistic regression, adjusted for potential confounders.	Longer sleep duration was associated with higher odds of periodontitis among women, especially in premenopausal women, but not among men. Compared with women sleeping ≤ 5 h, women sleeping 6–8 h had an adjusted OR of ~1.29 (95% CI: 1.06–1.56) and those sleeping ≥ 9 h had OR~1.45 (95% CI: 1.07–1.96) for periodontitis. In men, no significant relationship was observed between sleep duration and periodontitis after adjustment for confounders.
Islam et al. [49]	To evaluate the association between sleep quality and sleep duration in periodontitis affected university students	Cross-sectional study	1.934 participants	Participants from Okayama University (Japan) completed questionnaires on lifestyle, oral health, and sleep (PSQI-J). Dentists assessed periodontal status using PPD, BOP, and OHI-S, defining disease as PPD ≥ 4 mm and BOP ≥ 30%. Multivariable logistic regression was used to analyze associations between sleep and periodontal disease, adjusting for confounders.	Periodontal disease was present in 14.6% of students, and 19.2% had poor sleep quality. In adjusted models, sleep quality and sleep duration were not significantly associated with periodontal disease. Poorer oral hygiene (higher OHI-S) was the strongest predictor of periodontal disease.
Iwasaki et al. [34]	To evaluate the association between sleep duration and severe periodontitis in Japanese workers.	Cross-sectional study	1130 participants	Participants underwent full-mouth periodontal examinations and health check-ups and completed a self-administered questionnaire that included questions on sleep. Periodontitis was defined according to CDC/AAP criteria. Logistic regression and a restricted cubic spline model were used to analyze the data.	Severe periodontitis was identified in 6.3% of the study population. Those with <5, 5–5.9, 6–6.9, 7–7.9, and ≥8 h of sleep were 6.7%, 17.4%, 40.3%, 26.3%, and 8.9%, respectively. After adjusting for potential confounders, study participants who slept <5 h were more likely to have severe periodontitis than those who slept 7–7.9 h.
Jayachandran et al. [57]	To analyze the quality and duration of sleep and their potential association with periodontitis in the South Indian population.	Cross-sectional study	56 participants	Participants underwent full-mouth periodontal examinations. Periodontitis was diagnosed and classified according to the 2017 periodontal classification. They were given a questionnaire including demographic information and the PSQI.	There was a significant association between the severity and extent of periodontitis and sleep quality and duration. The mean sleep quality was highest in Stage IV (1.57 ± 0.76) and Grade B (1.62 ± 1.28). Similarly, Stage IV (1.14 ± 0.66) and Grade B (1.38 ± 0.92) showed the highest scores for sleep duration.
Kadim et al. [58]	To assess whether there is a relationship between periodontal diseases and sleep deficiency in a sample of patients from the College of Dentistry.	Cross-sectional study	45 participants	Participants were divided into three groups (n = 15 each): clinically healthy group, gingivitis group, and periodontitis group. Periodontal status was evaluated using PI, GI, and PPD. Sleep deficiency was assessed using the PSQI questionnaire.	The mean PSQI scores were statistically non-significant across all groups (Healthy, Gingivitis, and Periodontitis). Indicating an independent association of sleep deficiency with severity of periodontal disease.
Karaaslan et al. [50]	To investigate the association of stage-grade of periodontitis with sleep quality. and the effect of periodontitis on QoL.	Cross-sectional study	99 participants	Clinical examination and a questionnaire were administered to participants. The questionnaire was based on demographic information, OHIP-14, and PSQI. Patients were diagnosed according to the 2017 periodontal classification Clinical examination included PPD and CAL.	The mean clinical values of the patients in this study included CAL, 4.03 ± 2.46 mm, and PPD, 4.27 ± 1.55 mm. The mean of the global OHIP-14 score was 13.43 ± 6.23 and the mean PSQI global score was 6.57 ± 3.53. Stage-grade of periodontitis was associated with short sleep duration, low-sleep quality, and low oral health-related QoL.
Li et al. [62]	To investigate how sleep deficiency exacerbates periodontitis and to explore the underlying neuro–immune mechanisms, focusing on the role of trigeminal TRPV1 neurons.	Cross-sectional human and experimental animal study	6913 human participants, 17 mice	Clinical data from NHANES about correlation between sleep deficiency and increased periodontitis risk were combined with mouse models of experimental periodontitis under sleep restriction. Retrograde neuronal tracing from the *periodontium* was performed to identify trigeminal pathways, and neuronal ablation, pharmacological inhibition of substance P signaling with an NK1 receptor antagonist, and Tacr1 knockdown were used to assess functional outcomes.	Sleep deficiency was associated with increased risk and severity of periodontitis in both humans and mice. Trigeminal TRPV1-expressing neurons innervate periodontal tissues and, under sleep restriction, release more substance P, promoting vasodilation, vascular permeability, and inflammatory cell infiltration. Ablation of these neurons prevented the exacerbation of periodontitis induced by sleep deficiency, highlighting their key role.
Liu et al. [51]	To evaluate the association between sleep duration and the prevalence of periodontitis and tooth loss among U.S. adults.	A cross-sectional analysis of NHANES data.	5636 participants	NHANES data including U.S. adults aged ≥30 years were used. Sleep duration was self-reported and categorized as deficient, adequate, or excessive. Periodontitis was classified as none, mild, moderate, or severe based on the American Periodontal Association criteria. Tooth loss was determined through dental examination. Associations were analyzed using weighted multivariable logistic regression, GAM to explore nonlinear trends, and mediation analysis to identify indirect pathways.	Tooth loss was highly prevalent (96.4%), and 46.6% of participants had moderate to severe periodontitis. After adjusting for confounders, sleep deficiency was significantly associated with both moderate/severe periodontitis (OR 1.15, 95% CI 1.01–1.30) and tooth loss (OR 1.16, 95% CI 1.01–1.33). A U-shaped relationship was observed between sleep duration and periodontitis. Hypertension mediated about 7% of the association between sleep duration and tooth loss. Both insufficient and excessive sleep are independently linked to a higher risk of moderate/severe periodontitis and tooth loss.
Marruganti et al. [52]	To evaluate the association of perceived stress and poor sleep quality with periodontitis in a university-based cohort of individuals.	Cross-sectional study	235 participants aged 18–70 years with at least two remaining teeth.	Exposure variables were perceived stress, assessed with the Italian version of the PSS-10, and sleep quality, measured with the Italian PSQI. The presence and severity of periodontitis were assessed through full-mouth periodontal examination (PPD, REC, CAL) and classified according to both EFP/AAP and CDC/AAP case definitions. Statistical analysis included ordinal logistic regression (for periodontitis stages) and multiple linear regression (for mean CAL and PPD), adjusted for age, sex, smoking, BMI, education, oral hygiene habits, and comorbidities.	It was shown that compared with individuals with low stress and good sleep quality, those with moderate/high stress had an increased risk of stage III/IV periodontitis (OR ≈ 5.4; 95% CI 2.2–13.5; *p* < 0.001) and those with poor sleep quality had an OR ≈ 3.0 (95% CI 1.2–7.4; *p* < 0.05). The interaction between high stress and poor sleep quality had a multiplicative effect, with an OR ≈ 5.8 (95% CI 1.6–21.3; *p* < 0.001), and was also associated with worse mean CAL and PPD values.
Mehrizi et al. [64]	To compare the sleep quality of patients with periodontitis and their healthy counterparts.	Case–control study	106 patients with periodontitis and 106 controls	Participants were enrolled at the Periodontology Department of Yazd Dental School between December 2021 and April 2022. Periodontal status was assessed clinically using standard diagnostic criteria, while sleep quality was measured via PSQI. Demographic data and oral hygiene habits were collected using structured questionnaires. Statistical analyses, including t-tests, ANOVA, and multiple linear regression, were performed to evaluate associations between sleep quality and periodontal parameters.	Sleep quality scores were significantly worse in periodontitis patients compared with healthy controls, indicating poorer subjective sleep quality among those with periodontal disease. There was no significant correlation between sleep quality and age, gender, occupation, tooth-brushing pattern, or the severity of periodontal disease.
Nakada et al. [63]	To investigate the impact of sleep deprivation on periodontitis.	Experimental animal study	24 rats	Experimentally induced periodontitis was obtained by placing ligatures around the molars of the rats. Mice were divided into an experimental group with chronic sleep and a control group with normal sleep conditions. After the induction period, tissue samples were collected and analyzed histologically to CAL and alveolar bone resorption. Inflammatory markers were evaluated to assess both local and systemic inflammatory responses.	Rats subjected to sleep deprivation developed more severe periodontal destruction than those in the control group. They showed increased inflammatory infiltration in the periodontal tissues, greater CAL, and more pronounced alveolar bone resorption. The sleep-deprived animals also showed heightened systemic inflammation.
Park et al. [53]	To examine the association between shift work, sleep duration, and periodontal disease prevalence in a large adult population from South Korea.	Cross-sectional study	22,508 participants aged ≥ 19 years	Data of Korea KNHANES 2007–2012 were used to examine the correlation of sleep duration, shift work and periodontitis assessed according to CPI. Work of participants was categorized as day work vs. shift work; participants were divided into groups according to sleep duration: ≤ 5 h, 6–8 h, and ≥ 9 h per day. Periodontal status was measured using the CPI with CPI ≥ 3 defined as periodontitis. Analysis was multivariable logistic regression adjusted for confounders; an interaction term tested combined effects of work type and sleep duration.	Night shift work was associated with a significantly higher risk of periodontitis (OR ≈ 2.17). Sleep duration alone (short or long) was not linked to increased risk. However, when combined with shift work, both short and long sleep were associated with markedly higher odds of periodontitis; no increased risk was observed among day workers.
Romandini et al. [59]	To investigate whether sleep duration is associated with the prevalence of periodontitis in a general population.	Cross-sectional study	5812 adult participants	Participants underwent periodontal assessment using the CPO to define periodontitis. Sleep duration was self-reported and categorized into different groups (≤5, 6, 7, 8, and ≥9 h/day). Multivariate logistic regression analyses were performed to evaluate the association between sleep duration and periodontitis while adjusting for potential confounders.	A significant association between sleep duration and periodontitis prevalence was found. Compared to individuals sleeping ≤5 h/day, all other sleep duration groups showed higher odds of periodontitis, with the strongest association observed in those sleeping ≥9 h/day. The relationship appeared independent of major confounding factors and was more pronounced in certain subgroups. Periodontitis was associated independently with abnormal sleep duration.
Wiener et al. [60]	To evaluate whether routine inadequate sleep duration is associated with periodontitis in adults.	Observational cross-sectional study	3740 participants aged ≥30 years	Data were obtained from merged NHANES cycles (2009–2010 and 2011–2012). Periodontitis (yes/no) was the primary outcome, assessed through standardized clinical examination, while sleep duration on weekday/workday nights was self-reported and used as the main exposure variable. Statistical analyses included chi-square tests and multivariate logistic regression to evaluate the association between sleep duration and periodontitis, adjusting for potential confounders	Among participants, 52.7% had periodontitis and 35.7% reported sleeping less than 7 h per night. However, after adjustment, no statistically significant association was found between inadequate sleep duration (<7 h) and periodontitis (OR ≈ 1.00; *p* ≈ 0.98). Short sleep duration was not significantly linked to periodontitis in this nationally representative sample.
Yuan et al. [61]	To evaluate the association between sleep patterns (especially sleep duration) and periodontitis, and to further investigate whether this relationship is causal using Mendelian randomization.	Cross-sectional observational study combined with a Mendelian randomization analysis.	7289 participants	Periodontitis was assessed through clinical oral examinations, while sleep duration was obtained from survey data. The association between sleep and periodontitis was analyzed using logistic regression models, with additional stratified analyses by demographic variables. To explore causality, the authors conducted a Mendelian randomization analysis using publicly available genetic datasets related to sleep traits.	Short sleep duration (<7 h/night) was associated with a higher risk of periodontitis (OR ≈ 1.25), particularly in subgroups such as younger individuals, women, and those with lower education levels. However, the Mendelian randomization analysis found no causal relationship between genetically predicted sleep traits (sleep duration or insomnia) and periodontitis.

ANOVA: Analysis of Variance; BMI: Body Mass Index; BOP: Bleeding on Probing; CAL: Clinical Attachment Loss; CDC/AAP: Centers for Disease Control and Prevention/American Academy of Periodontology; CPI: Community Periodontal Index; EFP/AAP: European Federation of Periodontology/American Academy of Periodontology; GAM: Generalized Additive Models; GI: Gingival Index; NHANES: National Health and Nutrition Examination Survey; OHI-S: Oral Hygiene Index Simplified; OHIP-14: Oral Health Impact Profile-14; OR: Odds Ratio; PI: Plaque Index; PPD: Probing Pocket Depth; PSM: Propensity Score Matching; PSS-10: Perceived Stress Scale-10; PSQI: Pittsburgh Sleep Quality Index; PSQI-J: Pittsburgh Sleep Quality Index of Japan; QoL: Quality of Life; REC: Gingival Recession; TRPV1: Transient Receptor Potential Vanilloid 1; U.S.: United States; WBC: White Blood Cells.

## Data Availability

Data are available from the corresponding author upon reasonnable request.

## Supplementary Materials

The following supporting information can be downloaded at: https://www.mdpi.com/article/10.3390/medicina62040662/s1. File S1: PRISMA Checklist [88]. Table S1. Search Strategy for all the consulted databases.
