# Supplementary material for: The Association of Periodontitis with Circadian Rhythm Disorders and Sleep—A Scoping Review
Source: Medicina (Kaunas). 2026 Mar 31;62(4):662. doi: 10.3390/medicina62040662 (PMC13117668; doi:10.3390/medicina62040662)
Supplement: Supplementary file 1 [file Table S1.pdf › Table S1.pdf]

Scientific Databases	Search strategy	Hits
MEDLINE - PubMed searched on March 20, 2026 https://pubmed.ncbi.nlm.nih.gov/	(periodontitis OR "periodontal disease") AND ("circadian rhythm" OR melatonin OR cortisol OR "sleep disturbance" OR "sleep quality" OR "sleep duration" OR insomnia OR "sleep deprivation")	558
SCOPUS searched on March 20, 2026 www.scopus.com	(periodontitis OR "periodontal disease") AND ("circadian rhythm" OR melatonin OR cortisol OR "sleep disturbance" OR "sleep quality" OR "sleep duration" OR insomnia OR "sleep deprivation")	709
WEB OF SCIENCE searched on March 20, 2026 https://www.webofscience.comhttps	(periodontitis OR "periodontal disease") AND ("circadian rhythm" OR melatonin OR cortisol OR "sleep disturbance" OR "sleep quality" OR "sleep duration" OR insomnia OR "sleep deprivation")	549
Cochrane Library searched on March 20, 2026 ://www.cochranelibrary.com/	(periodontitis OR "periodontal disease") AND ("circadian rhythm" OR melatonin OR cortisol OR "sleep disturbance" OR "sleep quality" OR "sleep duration" OR insomnia OR "sleep deprivation")	108
**Total**		**1919**

Supplementary Table S1. Search Strategy for all the consulted databases.
